# Clipping versus coiling for aneurysmal subarachnoid hemorrhage: a systematic review and meta-analysis of prospective studies

**DOI:** 10.1007/s10143-021-01704-0

**Published:** 2021-12-06

**Authors:** Wenjun Zhu, Xiaoxiao Ling, Jindong Ding Petersen, Jinyu Liu, Anqi Xiao, Jiayan Huang

**Affiliations:** 1 School of Public Health, Fudan University, Shanghai, 200433 China; 2 Key Lab of Health Technology Assessment, National Health Commission of the People’s Republic of China, Shanghai, 200433 China; 3 Department of Statistical Science, University College London, London, WC1E 6BT UK; 4 Department of Clinical Epidemiology, Department of Clinical Medicine, Aarhus University, Aarhus, Denmark; 5 Research Unit for General Practice, Department of Public Health, University of Copenhagen, Copenhagen, Denmark

**Keywords:** Aneurysmal subarachnoid hemorrhage, Neurosurgical clipping, Endovascular coiling, Effectiveness, Safety, Meta-analysis

## Abstract

Neurosurgical clipping and endovascular coiling are both standard therapies to prevent rebleeding after aneurysmal subarachnoid hemorrhage (aSAH). However, controversy still exists about which is the optimal treatment. This meta-analysis aims to assess the effectiveness and safety of two treatments with high-quality evidence. Web of Science, Cochrane Library, EMBASE, Pubmed, Sinomed, China National Knowledge Infrastructure, and Wanfang Data databases were systematically searched on August 5, 2021. Randomized controlled trials (RCTs) and prospective cohort studies that evaluated the effectiveness and safety of clipping versus coiling in aSAH patients at discharge or within 1-year follow-up period were eligible. No restriction was set on the publication date. Meta-analyses were conducted to calculate the pooled estimates and 95% confidence intervals (CI) of relative risk (RR). Eight RCTs and 20 prospective cohort studies were identified. Compared to coiling, clipping was associated with a lower rebleeding rate at discharge (RR: 0.52, 95% CI: 0.29––0.94) and a higher aneurysmal occlusion rate (RR: 1.33, 95% CI: 1.19–1.48) at 1-year follow-up. In contrast, coiling reduced the vasospasm rate at discharge (RR: 1.45, 95% CI: 1.23–1.71) and 1-year poor outcome rate (RR: 1.27, 95% CI: 1.16–1.39). Subgroup analyses presented that among patients with a poor neurological condition at admission, no statistically significant outcome difference existed between the two treatments. The overall prognosis was better among patients who received coiling, but this advantage was not significant among patients with a poor neurological condition at admission. Therefore, the selection of treatment modality for aSAH patients should be considered comprehensively.

**Supplementary Information:**

The online version contains supplementary material available at 10.1007/s10143-021-01704-0.

## Introduction

Intracranial aneurysm (IA) is a cerebrovascular disorder in which the weakness of a cerebral artery wall causes a localized dilation of the blood vessel. IA can develop and rupture, and about 85% of spontaneous subarachnoid hemorrhage (SAH) cases are caused by the rupture of IA [35]. The attack rate of SAH varied across countries. The estimated incidence was 19.7 and 22.7 per 100,000 person-year in high incidence regions—Finland and Japan, while in other regions the overall rate was 9.1 per 100,000 person-year [9].

The prognosis of aneurysmal SAH is poor. Acute hydrocephalus occurred among around 15–20% of IA patients, which could cause intense headaches [18]. Cerebral vasospasm can also appear within 3 to 12 days after SAH [5], which may lead to ischemic cerebral infarction and even death. Rebleeding is another major cause of death. Without invasive therapy, the cumulative rebleeding rate would reach approximately 19% over the ensuing 2 weeks [24]. Moreover, 10–20% of patients would have a severe disability, and one-third of them would die because of SAH [21].

To prevent the rebleeding and improve the capacity for independent living of patients, effective interventions are required. Two treatments are available globally: neurosurgical clipping and endovascular coiling. Clipping is a method that requires craniotomy under general anesthesia. Permanent clips are placed across the neck of the aneurysm to exclude the aneurysm from circulation [5]. Coiling is performed under the angiographic techniques. Detachable coils of different shapes and sizes are deposited into the aneurysm through a microcatheter, which reduces the blood flow and induces thrombus formation [33]. There is a lot of controversy on which of these two methods is optimal. Compared to coiling, clipping has better durability and can significantly reduce the retreatment rate, whereas it may cause stronger cerebral blood flow change in the nearby regions and a worse prognosis [6, 47]. On the other side, patients who have received coiling can benefit from minimally invasive surgery and faster recovery [23].

Several meta-analyses that compared the effectiveness of clipping and coiling have been published. They either only included randomized controlled trials (RCTs) [29, 45] or were based on evidence from RCTs and observational studies [14, 31]. Since RCTs have a high requirement on the eligibility of patients, the generalization of results is questionable [10]. For meta-analysis based on RCTs and observational studies, the reliability of cross-sectional and case–control studies is limited, and the combination of these two types of studies may reduce the internal validity of conclusions. Considering both internal and external validity, this systematic review aims to assess the effectiveness and safety of clipping compared with coiling based on high-quality evidence from both RCTs and prospective cohort studies, and to make a supplementary suggestion on the treatment of aneurysmal SAH patients.

## Methods

### Search strategy

A systematic search of Pubmed, Web of Science, EMBASE, Cochrane Library, the China National Knowledge Infrastructure (CNKI), Wanfang Data, and Sinomed was performed on August 5, 2021. The latter three are Chinese databases. The search strategies were presented in Supplemental Table 1. No restriction was set on the publication date. All references of included studies were also scanned to identify additional relevant articles.

**Table 1:** Detailed characteristics of the included studies

Study	Country	Study design	Sample size (clip/coil)	Age range	Time interval between SAH and treatment	Sex (M/F)	Anterior/posterior circulation	WFNS		H&H				
								1–3	4–5	1	2	3	4	5
Darsaut et al. (2019) [8]	Multinational	RCT	55/48	-	-	35/68	98/5	82	18	-	-	-	-	-
ISAT (2002) [39, 40, 44]	Multinational	RCT	1070/1073	18–87 y	0–28 d	798/1345	2085/58	2018	94	-	-	-	-	-
Li et al. (2012) [32]	China	RCT	92/94	-	-	130/56	185/1	-	-	117		53	16	
Vapalahti et al. (2000) [27, 54]	Finland	RCT	57/52	14–75 y	-	51/58	98/11	-	-	67		26	16	
Xu et al. (2018) [57]	China	RCT	30/30	-	-	34/26	-	-	-	13	27	16	4	0
Liu et al. (2007) [34]	China	RCT	44/30	24–78 y	-	41/33	69/5	-	-	39		26	9	
Li et al. (2015) [30]	China	RCT	38/38	32–76 y	-	56/20	-	-	-	-	-	-	-	-
Wan et al. (2017) [55]	China	RCT	43/40	65–85 y	-	49/34	-	-	-	-	-	-	-	-
Birski et al. (2014) [3]	Poland	PC	69/17	21–78 y	-	-	-	-	-	-	-	-	-	-
Hirohata et al. (2004) [20]	Japan	PC	101/179	-	0–72 h	75/205	239/41	-	-	0	227		53	
Shimauchi-Ohtaki et al. (2018) [46]	Japan	PC	16/14	-	-	8/22	28/2	21	9	-	-	-	-	-
Teo et al. (2017) [52]	Multinational	PC	254/513	20–69 y	0–17 d	242/525	695/72	591	176	-	-	-	-	-
Zhao et al. (2016) [59]	China	PC	129/133	–	0–21 d	131/131	235/27	0	262	-	-	-	-	-
Flett et al. (2005) [15]	England	PC	67/46	26–87 y	–	-	92/21	94	17	-	-	-	-	-
Suzuki et al. (2013) [50]	Japan	PC	282/297	-	0–12 d	187/392	502/77	385	194	-	-	-	-	-
Hammer et al. (2016) [17]	Germany	PC	390/271	-	0–48 h	258/403	597/64	468	193	-	-	-	-	-
Kawabata et al. (2011) [25]	Japan	PC	77/25	31–88 y	0–24 h	40/62	99/3	-	-	49		16	37	
Proust et al. (2009) [42]	France	PC	36/14	18–75 y	0–72 h	25/25	50/0	-	-	31		19		0
Dehdashti et al. (2004) [11]	Switzerland	PC	72/26	20–78 y	0–72 h	35/63	87/11	79	19	-	-	-	-	-
Kawai et al. (2008) [26]	Japan	PC	14/16	32–79 y	-	10/20	-	-	-	-	-	-	-	-
Dehdashti et al. (2004) [12]	Switzerland	PC	180/65	18–78 y	0–14 d	88/157	-	196	49	-	-	-	-	-
Koyanagi et al. (2019) [28]	Japan	PC	136/136	-	0–72 h	52/220	266/6	186	86	-	-	-	-	-
Mortimer et al. (2016) [41]	Australia	PC	66/69	-	0–72 h	42/93	113/22	96	39	-	-	-	-	-
Bian et al. (2012) [2]	China	PC	12/9	-	-	11/10	20/1	20	1	-	-	-	-	-
Groden et al. (2000) [16]	Germany	PC	12/22	10–78 y	0–60 d	-	0/34	-	-	9	2	14	7	2
Zhou et al. (2021) [60]	China	PC	68/80	-	-	79/69	113/35	-	-	-	-	-	-	-
Proust et al. (2020) [43]	France	PC	54/208	> 70 y	-	46/216	236/26	219	43	-	-	-	-	-
Wong et al. (2021) [56]	Canada	PC	95/287	-	-	116/266	325/57	287	95	-	-	-	-	-

*ISAT* the International Subarachnoid Aneurysm Trial, *RCT* randomized controlled trials, *PC* prospective cohort, *y* years old, *d* days, *M* male, *F* female, *WFNS* The World Federation of Neurosurgical Societies scale, *H&H* Hunt and Hess scales,—missing data

### Selection criteria

The selection process was undertaken by two reviewers independently, and any discrepancy was resolved by discussion. Studies were included if they met the following criteria: (1) RCTs and prospective cohort studies that compared clipping versus coiling in all age groups of aneurysmal SAH patients; (2) studies that reported at least one of the interested effectiveness or safety outcomes of patients at discharge or 1-year follow-up in the two groups. The effectiveness measures included poor outcome rate, mortality, and the rate of complete aneurysmal occlusion. Particularly, poor outcome was defined as death or dependence in daily activities (modified Rankin scale (mRS) with a score of 3–6 or Glasgow Outcome Scale (GOS) with a score of 1–3). The safety outcomes included the rate of rebleeding, ischemic cerebral infarction, vasospasm, and shunt-dependent hydrocephalus.

The exclusion criteria were as follows: (1) RCTs that did not report randomization methods; (2) prospective cohort studies with a substantial imbalance of preoperative characteristics or absence of baseline information; (3) SAH from trauma or infected aneurysms; (4) studies that did not present enough information for us to extract or calculate the absolute number of clinical events; (5) case reports, editorials, conference abstracts, comments, letters, and reviews. If the same data were used in more than one paper, the paper with the largest number of participants would be included.

### Data extraction

The following data were independently extracted by two reviewers using Microsoft Excel 2016, and any disagreement was discussed: (1) Study characteristics: journal, first author and his/her institution, publication year, study period, study setting, and study design. (2) Participants’ characteristics: study eligibility criteria, the age range, sex distribution, preoperative grade (including the World Federation of Neurosurgical Surgeons scale (WFNS), Hunt and Hess scales (H&H), and Fisher grade), aneurysm location, and aneurysm size of patients. (3) The number of patients treated with clipping or coiling, and outcomes of interest.

### Quality assessment

The methodological quality of RCTs was assessed by the Cochrane Risk of Bias Tool [19], which contains 7 domains: random sequence generation, allocation concealment, blinding of participants and personnel, blinding of outcome assessment, incomplete outcome data, selective reporting, and other biases. For other biases, the baseline characteristic balance between two groups, mainly including preoperative grades, age, and the time interval between SAH and treatment were assessed [49]. Each domain was scored as high, unclear, or low risk of bias.

The Newcastle–Ottawa scale was used to evaluate the quality of prospective cohort studies based on the selection bias, comparability of the exposed and unexposed cohorts, and outcome assessment of the studies [58]. The maximum score on the scale is 9 which indicates the highest quality.

Two reviewers assessed the quality of the selected studies separately using Review Manager 5.3 (Nordic Cochrane Centre, Cochrane Collaboration, Copenhagen, Denmark) and Microsoft Excel 2016. Any discrepancy was resolved by discussion.

### Statistical analysis

The relative risk (RR, clipping versus coiling) and 95% confidence interval (CI) of each study were estimated, and the results were presented in forest plots using Review Manager 5.3. For data synthesis, the *I*^2^ statistic, which indicates the proportion of total variation attributable to the variation between studies, was estimated to assess heterogeneity [19]. After that, the Mantel–Haenszel method was used to pool the data [13, 36]. The random-effect model would be used if *I*^2^ > 50%, which demonstrated a high level of heterogeneity. Otherwise, the fixed-effect model would be chosen.

Besides, if the outcomes of patients with specific characteristics were reported, subgroup analyses would be performed to examine which treatment was more suitable for these patients. A funnel plot would also be presented to examine the potential publication bias if more than 10 studies within the same comparison were included [19]. All the findings were reported based on the PRISMA Checklist [38].

## Results

### Results of the search

**Fig. 1:**
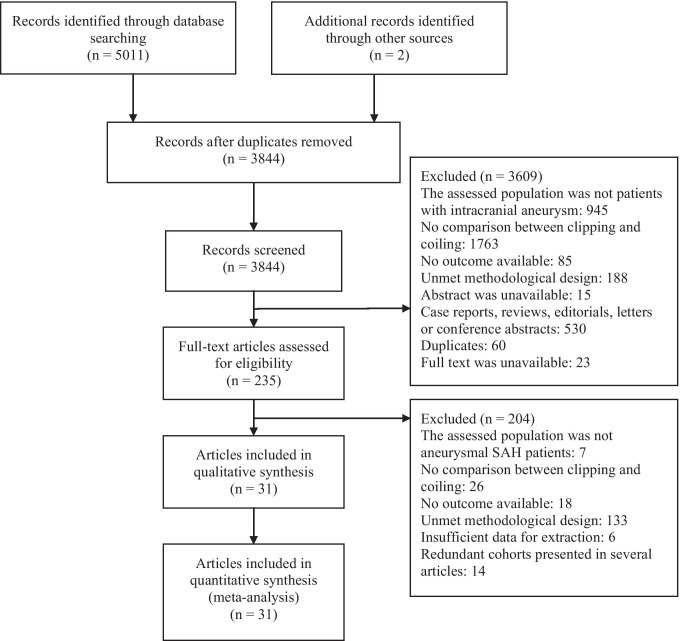
Flow chart of the selection procedure. SAH subarachnoid hemorrhage

The literature search identified 5011 articles. Two articles were also found after screening the reference of the included studies. After the removal of duplicates, 3844 studies were screened by titles and abstracts, and 3609 of them were excluded. A total of 235 full-text articles were retrieved, and 204 of them were excluded. Among them, the most common exclusion reason was that RCTs did not report randomization methods or prospective cohort studies did not report baseline information. Eventually, 31 articles were included in this meta-analysis. Among them, 27 articles were written in English, while others were in Chinese. The detailed process of selection and the reasons for exclusion are shown in Fig. 1.

Three articles reported the short-term or 1-year follow-up outcome from the International Subarachnoid Aneurysm Trial (ISAT) study [39, 40, 44], and an RCT conducted by Kuopio University Hospital also published two related articles [27, 54]. Other 26 articles described the results from 6 RCTs and 20 prospective cohort studies respectively. As a result, we included 28 independent studies. These studies enrolled 7391 patients in total, with 3559 patients who went through clipping and 3832 patients who received coiling. The median sample size was 111 patients (range, 21–2143 patients), and the age range of enrolled patients was 10 to 88 years old. The median proportion of patients whose aneurysms were located in the anterior circulation was 90.20% (range, 0.00–100.0%), and the median proportion of patients with the WFNS classification of 1–3 was 77.05% (range, 0.00%-95.55%).

Fourteen studies were conducted in Asia, 10 in Europe, and 1 in Australia, while the other 3 studies were multinational. A detailed description of the included studies is shown in Table 1.

### The methodological quality of the included studies

**Fig. 2:**
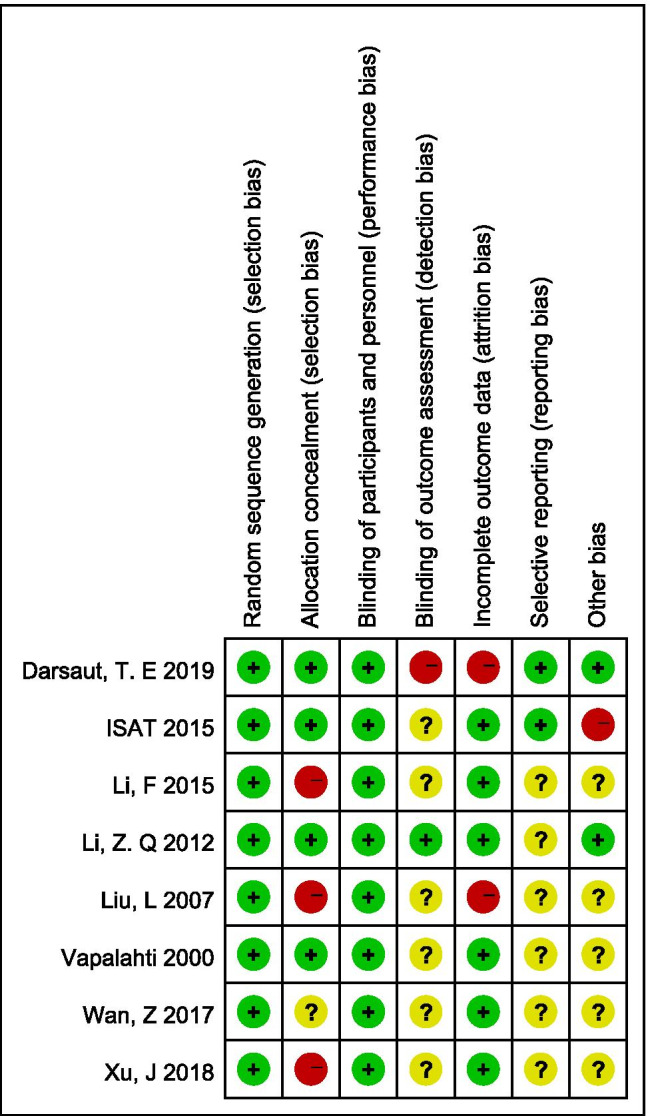
The detailed methodological quality of included RCTs. ISAT the International Subarachnoid Aneurysm Trial

Cochrane Risk of Bias Tool was applied to assess the methodological quality of the RCTs. In all, among the 8 RCTs included, 4 studies clearly described the practice of allocation concealment [8, 27, 32, 39, 40, 44, 54], and 6 studies did not have any loss to follow-up and thus reported all outcome data [27, 30, 32, 39, 40, 44, 54, 55, 57]. In terms of blinding, it is impossible to blind the surgeons, patients, or caregivers given the consideration of ethical and technical issues. However, it has been found that the un-blindness would introduce low bias since both clipping and coiling are standard treatment methods for SAH. The blinding of outcome assessment is feasible, but it was reported in only 1 study [32]. Two studies published the protocol [8, 39, 40, 44], and the reporting bias of the remaining studies was unclear. Besides, 5 studies did not report the baseline characteristics of patients thoroughly [27, 30, 34, 54, 55, 57]. The results of the methodological quality of RCTs are shown in Fig. 2 and Supplemental Fig. 1.

The overall methodological quality of cohort studies included was relatively high, as 70.00% of them earned ≥ 8 scores. The representativeness of the cohort was low in 5 studies because they did not include consecutive or random samples [2, 16, 28, 52, 60]. Seven studies did not state clearly how they assessed the outcomes [3, 12, 16, 25, 46, 56, 60]. The methodological scores of each cohort are presented in Table 2.

**Table 2:** The methodological scores of each cohort

Study	Representativeness of the exposed	Selection of the non-exposed	Ascertainment of exposure	Outcome was not present at start	Comparability	Assessment of outcome	Long enough follow-up	Adequacy of follow-up	Total
Birski et al. (2014) [3]	1	1	1	1	1	0	1	1	7
Hirohata et al. (2004) [20]	1	1	1	1	1	1	1	1	8
Shimauchi-Ohtaki et al. (2018) [46]	1	1	1	1	2	0	1	1	8
Teo et al. (2017) [52]	0	1	1	1	1	1	1	1	7
Zhao et al. (2016) [59]	1	1	1	1	1	1	1	1	8
Flett et al. (2005) [15]	1	1	1	1	2	1	1	1	9
Suzuki et al. (2013) [50]	1	1	1	1	1	1	1	1	8
Hammer et al. (2016) [17]	1	1	1	1	2	1	1	1	9
Kawabata et al. (2011) [25]	1	1	1	1	2	0	1	1	8
Proust et al. (2009) [42]	1	1	1	1	2	1	1	1	9
Dehdashti et al. (2004) [11]	1	1	1	1	2	1	1	1	9
Kawai et al. (2008) [26]	1	1	1	1	1	1	1	1	8
Dehdashti et al. (2004) [12]	1	1	1	1	2	0	1	1	8
Koyanagi et al. (2019) [28]	0	1	1	1	2	1	1	1	8
Mortimer et al. (2016) [41]	1	1	1	1	2	1	1	1	9
Bian et al. (2012) [2]	0	1	1	1	2	1	1	1	8
Groden et al. (2000) [16]	0	1	1	1	2	0	1	1	7
Zhou et al. (2021) [60]	0	1	1	1	2	0	1	1	7
Proust et al. (2020) [43]	1	1	1	1	1	1	1	0	7
Wong et al. (2021) [56]	1	1	1	1	1	0	1	0	6

### Effectiveness outcome

**Fig. 3:**
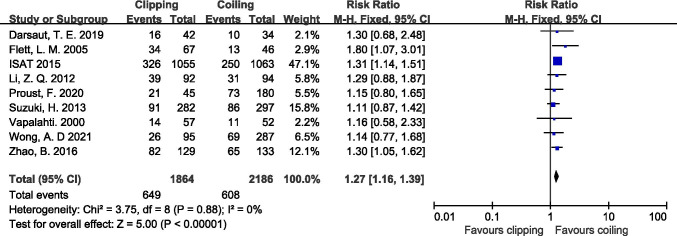
Effect of clipping versus coiling on the poor outcome rate at 1-year follow-up. ISAT the International Subarachnoid Aneurysm Trial, CI confidence interval, M-H Mantel–Haenszel method

Eight studies with a total of 2167 participants reported the poor outcome at discharge [3, 8, 20, 43, 46, 52, 56, 59]. The result indicated that the risk of poor outcome for patients who went through clipping was 1.22 times higher compared with patients who accepted coiling (RR: 1.22, 95% CI: 1.00–1.47, *P* = 0.04, Supplemental Fig. 2). Nine studies reported the 1-year outcome of 4050 participants [8, 15, 27, 32, 40, 43, 50, 56, 59], and results showed that clipping was associated with a 27% greater risk of poor outcome compared with coiling (RR:1.27, 95% CI: 1.16–1.39, *P* < 0.001, Fig. 3).

Six studies reported mortality at discharge [3, 8, 20, 46, 56, 59]. The pooled result of 1138 patients showed that the mortality did not differ significantly between the two treatment groups at discharge (RR: 0.94, 95% CI: 0.69–1.28, *P* = 0.69, Supplemental Fig. 3). No significant difference was found for 1-year mortality based on ten studies (RR: 1.07, 95% CI: 0.91–1.26, *P* = 0.44, Supplemental Fig. 4) [8, 15, 17, 27, 32, 40, 43, 56, 57, 59].

**Fig. 4:**
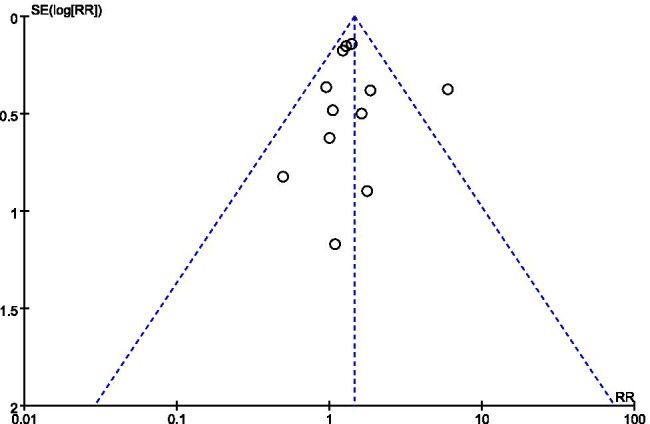
Publication bias of included studies on the vasospasm rate at discharge. SE standard error, RR relative risk

Four studies evaluated complete aneurysmal occlusion in 894 participants at discharge and found that clipping was superior to coiling (RR: 1.21, 95% CI: 1.15–1.28, *P* < 0.001, Supplemental Fig. 5) [17, 34, 42, 54]. At 1-year follow-up, clipping could increase the incidence of complete aneurysmal occlusion by 33% compared to coiling (RR: 1.33, 95% CI: 1.19–1.48, *P* < 0.001, Supplemental Fig. 6) [8, 15, 27, 32, 57].

### Safety outcome

Eight studies with 1868 patients reported the rebleeding rate at discharge [8, 17, 20, 25, 42, 43, 59, 60]. Consequently, clipping was associated with an approximately 48% decrease in the risk of rebleeding compared to coiling (RR: 0.52, 95% CI: 0.29–0.94, *P* = 0.03, Supplemental Fig. 7). But this advantage was no longer held at 1-year follow-up (RR: 0.56, 95% CI: 0.22–1.40, *P* = 0.21, Supplemental Fig. 8) according to the pooled analysis from 5 studies [15, 17, 32, 40, 56].

Nine studies assessed ischemic cerebral infarction at discharge including 2076 participants [11, 17, 20, 26, 30, 46, 50, 57, 59] and showed no significant effect difference between the two groups (RR: 1.09, 95% CI: 0.59–2.03, *P* = 0.78, Supplemental Fig. 9). Four studies reported a 1-year follow-up outcome [8, 17, 32, 56], and no significant difference was found (RR: 1.15, 95% CI: 0.54–2.44, *P* = 0.72, Supplemental Fig. 10).

In terms of other complications, 8 studies reported shunt-dependent hydrocephalus at discharge, and the pooled analysis showed no significant difference between the two groups (RR: 1.06, 95% CI: 0.68–1.67, *P* = 0.79, Supplemental Fig. 11) [8, 12, 28, 30, 41, 46, 57, 59]. Furthermore, the pooled summary of 12 studies showed a 45% increase in the risk of vasospasm at discharge if patients received clipping (RR: 1.45, 95% CI: 1.23–1.71, *P* < 0.001, Supplemental Fig. 12) [2, 8, 11, 16, 20, 41, 46, 50, 55, 57, 59, 60].

The effectiveness and safety outcomes of the two treatment arms are summarized in Table 3.

**Table 3:** The effectiveness and safety outcomes of the two treatments

Category	Indicator	*N*	RR	95% CI	Favors clipping	Favors coiling	No significant difference
Effectiveness	Poor outcome—discharge	8	1.22*	1.00–1.47		√	
	Poor outcome—1 year	9	1.27*	1.16–1.39		√	
	Mortality—discharge	6	0.94	0.69–1.28			√
	Mortality—1 year	10	1.07	0.91–1.26			√
	Complete aneurysmal occlusion—discharge	4	1.21*	1.15–1.28	√		
	Complete aneurysmal occlusion—1 year	5	1.33*	1.19–1.48	√		
Safety	Rebleeding—discharge	8	0.52*	0.29–0.94	√		
	Rebleeding—1 year	5	0.56	0.22–1.40			√
	Ischemic cerebral infarction—discharge	9	1.09	0.59–2.03			√
	Ischemic cerebral infarction—1 year	4	1.15	0.54–2.44			√
	Shunt-dependent hydrocephalus—discharge	8	1.06	0.68–1.67			√
	Vasospasm—discharge	12	1.45*	1.23–1.71		√	

*N* num of studies, *RR* relative risk, *CI* confidence interval

^*^p < 0.05

### Subgroup analyses

Several studies reported the 1-year poor outcome of subgroups (Supplemental Fig. 13) [15, 27, 39, 40, 43]. It was suggested that, among patients with a poor neurological condition at admission (WFNS of 4–6), there was no statistically significant difference between clipping and coiling groups (RR: 1.02, 95% CI: 0.82–1.27, *P* = 0.84). Also, the outcome was not significantly different between the two treatment groups in patients with the anterior cerebral artery, anterior communicating artery (ACA-AComA) (RR: 1.12, 95% CI: 0.92–1.37, *P* = 0.26), or middle cerebral artery (MCA) aneurysms (RR: 0.98, 95% CI: 0.69–1.40, *P* = 0.92). However, coiling yielded a better outcome for patients with the internal carotid artery (ICA) (RR: 1.76, 95% CI: 1.37–2.25, *P* < 0.001) or posterior circulation artery (PCA) aneurysms (RR: 2.41, 95% CI: 1.08–5.37, *P* = 0.03). The results of the subgroup analyses are summarized in Table 4.

**Table 4:** The results of subgroup analyses

Indicator	Subgroup	*N*	RR	95% CI	Favors clipping	Favors coiling	No significant difference
Poor outcome—1 year	Fisher grade of 0–2	2	1.63*	1.06–2.48		√	
	Fisher grade of 3–4	2	1.26*	1.09–1.45		√	
	WFNS of 1–3	3	1.40*	1.21–1.62		√	
	WFNS of 4–6	3	1.02	0.82–1.27			√
	ACA-AComA	2	1.12	0.92–1.37			√
	MCA	2	0.98	0.69–1.40			√
	ICA	2	1.76*	1.37–2.25		√	
	PCA	2	2.41*	1.08–5.37		√	

*N* num of studies, *RR* relative risk, *CI* confidence interval, *WFNS* the World Federation of Neurosurgical Surgeons scale, *ACA* anterior cerebral artery, *AComA* anterior communicating artery, *MCA* middle cerebral artery, *ICA* internal carotid artery, *PCA* posterior circulation artery

^*^p < 0.05

### Publication bias

Only the comparison of the vasospasm rate included more than 10 studies, and the funnel plot implied the existence of publication bias (Fig. 4).

## Discussion

In all, according to our meta-analysis, clipping had advantages in occluding aneurysms more completely and reducing the risk of rebleeding. In comparison, coiling could lead to a risk reduction for poor outcome and vasospasm. The mortality, ischemic cerebral infarction rate, and shunt-dependent hydrocephalus rate did not differ significantly between the two groups. The updated Cochrane systematic review which only included 4 RCTs demonstrated a better outcome for coiling patients despite its higher rebleeding rate, which is in line with our results [33]. However, since its results were largely influenced by ISAT, the evidence of the Cochrane review could only be extrapolated to patients with a better health status or aneurysms located in the anterior circulation. The meta-analysis conducted by Li et al. included 4 RCTs, 7 prospective cohort studies, 14 retrospective cohort studies, and 2 ambispective cohort studies [31]. It also found that clipping reduced the incidence of rebleeding, which could be explained by its better complete occlusion rate, and there was no significant difference for mortality. Vasospasm was also more common among patients treated with clipping. Besides, a nationwide database-based meta-analysis also found that in-hospital mortality was not significantly different between matched clipping and coiling groups [22].

According to the European Stroke Organization (ESO) Guidelines, the neurological condition of SAH patients when they were admitted has a strong influence on their prognosis. This condition is generally assessed by several grading instruments, such as WFNS, H&H, and Glasgow Coma Scale [49]. This enables us to undertake subgroup analyses to test whether the effects of the two treatments were similar for patients with different preoperative grades. The results demonstrated that among patients with a poor neurological condition at admission, treatment modality was not a significant prognostic factor. Comparable conclusions have also been drawn by Li et al. [31]. Besides, our subgroup analyses also discovered that the prognosis was not significantly affected by treatments in patients with ACA, AComA, or MCA aneurysms. ESO Guidelines also recommended that patients with MCA should preferably be treated by clipping [49]. However, since each subgroup analysis only included 2–3 studies, the evidence was relatively weak. Further RCTs and prospective cohort studies are needed to investigate the treatment effectiveness for patients with different characteristics.

Currently, coiling has become the dominant treatment method in many countries, such as the USA, western Europe, and China due to the ISAT trial [1, 4, 48]. Also, as craniotomy is more difficult to operate than minimally invasive surgery, the spread of coiling treatment is more rapid than clipping. However, our subgroup analyses suggested that the decision on treatment selection should be made based on the clinical characteristics of SAH patients. Besides, as the medical costs of coiling are generally higher than clipping [37, 51], “coiling mainly” policy may impose an additional or unnecessary economic burden on both society and patients. Therefore, in consideration of both clinical benefits and health expenses, the preference for coiling should be changed and clinical guidelines should be considered. So far, both ESO and the American Heart Association/American Stroke Association have published guidelines for the management of patients with ruptured and unruptured IA [49, 53]. Countries can use and amend them based on the health needs of the local population and the level of medical technology.

This review combined the evidence generated by RCTs and prospective cohort studies. Compared to the current RCT-based systematic review [33], we included other four RCTs that were conducted in China and published in Chinese journals. In addition to this, the recently published interim analysis of the ISAT-2 trial, which considered patients that were not included in the ISAT trial [7], has been added. This evidence may be helpful to improve the representativeness of included patients. Moreover, the overall methodological quality of the eligible cohort studies was high, considering both the representativeness and baseline comparability. Therefore, the results of this review are pragmatic in routine clinical practice.

This study has the following limitations: First, only two RCTs published the research protocols, and reporting bias of other RCTs remains unclear. Besides, 62.50% of included RCTs did not provide enough information on baseline characteristics of patients, and 40.00% of cohort studies did not reach a complete balance in baseline characteristics between clipping and coiling cohorts. The uncertainty and imbalance between the two treatment groups could introduce confounder bias. Second, most of the studies were performed in Europe, Eastern Asia, and North America. The results may be unrepresentative to other regions of the world.

## Conclusion

In summary, each treatment modality has its own pros and cons. Coiling yielded a better clinical outcome at short- and long-term follow-up, but the rebleeding risk was lower if patients received clipping. Besides, for patients with a poor neurological condition at admission, there was no statistically significant outcome difference between the two treatments. Therefore, comprehensive considerations should be given on the selection of treatment for SAH patients, considering both patients’ preference and their preoperative condition.

## Supplementary Information

Below is the link to the electronic supplementary material.

Supplementary file1 (PDF 409 kb)

## Data Availability

Not applicable.
