# Supplementary material for: Clipping versus coiling for aneurysmal subarachnoid hemorrhage: a systematic review and meta-analysis of prospective studies
Source: Neurosurg Rev. 2021 Dec 6;45(2):1291–302. doi: 10.1007/s10143-021-01704-0 (PMC8976818; doi:10.1007/s10143-021-01704-0)
Supplement: Supplementary file 1 — Supplementary file1 (PDF 409 kb) [file 10143_2021_1704_MOESM1_ESM.pdf]

Clipping versus coiling for aneurysmal subarachnoid hemorrhage: a systematic review and meta-analysis of prospective studies

Journal name: Neurosurgical review

Wenjun Zhu[1,2], XiaoXiao Ling[3], Jindong Ding Petersen[4,5], Jinyu Liu[1,2], Anqi Xiao[1,2], Jiayan Huang[1,2*]

1 School of Public Health, Fudan University, Shanghai 200433, China

2 Key Lab of Health Technology Assessment, National Health Commission of the People's Republic of China, Shanghai 200433, China

3 Department of Statistical Science, University College London, London WC1E 6BT, United Kingdom

4 Department of Clinical Epidemiology, Department of Clinical Medicine, Aarhus University, Denmark

5 Research Unit for General Practice, Department of Public Health, University of Copenhagen, Copenhagen, Denmark

Correspondence to:

Jiayan Huang, Key Lab of Health Technology Assessment, National Health Commission; School of Public Health, Fudan University, Shanghai, 200433, China

jiayanhuang@fudan.edu.cn

Supplemental Table 1: Search strategy.

Search set	Embase	Items found
#1	exp intracranial aneurysm/	
#2	exp brain artery aneurysm/	
#3	(intracranial or cerebral or Brain or "Basilar Artery" or Saccular or "Anterior Communicating Artery" or "Posterior Communicating Artery" or "Middle Cerebral Artery" or "Posterior Cerebral Artery" or "Posterior Cerebral Artery" or "Anterior circulation" or "Posterior circulation" or Berry or subarachnoid).ab,kw,ti.	
#4	(aneurysm* or hemorrhage*).ab,kw,ti.	
#5	#3 and #4	
#6	exp subarachnoid hemorrhage/	
#7	#1 or #2 or #5 or #6	
#8	exp craniotomy/	
#9	exp craniectomy/	
#10	exp aneurysm clip/	
#11	exp microsurgery/	
#12	(Craniotom* or Craniectom* or clip* or microsurg*).ab,kw,ti.	
#13	#8 or #9 or #10 or #11 or #12	
#14	exp coil embolization/	
#15	exp endovascular aneurysm repair/ or exp endovascular surgery/	
#16	exp neuroradiology/	
#17	exp prosthesis/	
#18	exp prosthesis/	
#19	exp implant/	
#20	exp endoprosthesis/	
#21	(coil* or emboli* or Embolotherap* or endovascular or Intravascular or neuroradiology or Guglielmi or Prosthes* or Implant* or Endoprosthes*).ab,kw,ti.	
#22	#14 or #15 or #16 or #17 or #18 or #19 or #20 or #21	
#23	exp death/	
#24	exp morbidity/	
#25	exp mortality/	
#26	exp fatality/	
#27	exp dependent personality disorder/	
#28	exp disability/	
#29	exp treatment outcome/ or exp clinical outcome/ or exp Glasgow outcome scale/	
#30	exp comparative effectiveness/	
#31	exp prognosis/	
#32	exp cerebrovascular accident/ or exp stroke/ or exp brain ischemia/ or exp Rankin scale/	
#33	exp complication/	
#34	exp brain vasospasm/	
#35	exp infarction/	
#36	exp brain infection/	
#37	exp hydrocephalus/	
#38	exp recanalization/	
#39	exp hospitalization/	

#40	(Death or Mortalit* or Fatalit* or Morbidit* or Dependenc* or Disabl* or outcome or Efficacy or Effectiveness or Prognos* or "Glasgow Outcome Scale" or gos or "Modified Rankin Score" or mrs or Complication* or Vasospasm* or Angiospasm* or "Vascular Spasm*" or "Cerebrovascular Spasm*" or Ischemi* or Infarct* or Stroke* or "Intracranial infection" or Hydrocephal*).ab,kw,ti.	
#41	(Rebleed or Obliteration* or Occlusion* or Recanalisation or "Hospital stay*" or "Length of Stay" or "Stay Length*" or (Neurologic* and (deficit* or Symptom* or Sign or Dysfunction* or Manifestation*))).ab,kw,ti.	
#42	#23 or #24 or #25 or #26 or #27 or #28 or #29 or #30 or #31 or #32 or #33 or #34 or #35 or #36 or #37 or #38 or #39 or #40 or #41	
#43	("Randomized Controlled" or "Randomized Clinical" or "Controlled Clinical" or RCT or Cohort or "Concurrent Stud*" or Prospect*).ab,kw,ti.	
#44	exp randomized controlled trial/	
#45	exp clinical trial/ or exp controlled study/	
#46	exp cohort analysis/	
#47	exp prospective study/	
#48	#43 or #44 or #45 or #46 or #47	
#49	#7 and #13 and #22 and #42 and #48	2352
	Web of science	
#1	TS=((intracranial OR cerebral OR Brain OR "Basilar Artery" OR Saccular OR "Anterior Communicating Artery" OR "Posterior Communicating Artery" OR "Middle Cerebral Artery" OR "Posterior Cerebral Artery" OR "Anterior Cerebral Artery" OR "Anterior circulation" OR "Posterior circulation" OR Berry OR subarachnoid) AND (aneurysm* OR hemorrhage*))	
#2	TS=(Craniotom* OR Craniectom* OR clip* OR microsurg*)	
#3	TS=(coil* OR emboli* OR Embolotherap* OR endovascular OR Intravascular OR neuroradiology OR Guglielmi OR Prosthes$s OR Implant$ OR Endoprosthes$s)	
#4	TS=(Death OR Mortalit* OR Fatalit* OR Morbidit* OR Dependenc* OR Disabl* OR outcome OR Efficacy OR Effectiveness OR Prognos* OR "Glasgow Outcome Scale" OR gos OR "Modified Rankin Score" OR mrs OR Complication* OR Vasospasm$ OR Angiospasm$ OR "Vascular Spasm$" OR "Cerebrovascular Spasm$" OR Ischemi* OR Infarct* OR Stroke$ OR "Intracranial infection" OR Hydrocephal* OR Rebleed OR Obliteration$ OR Occlusion$ OR Recanalisation OR "Hospital stay$" OR "Length of Stay" OR "Stay Length$" OR (Neurologic* AND (deficit$ OR Symptom$ OR Sign$ OR Dysfunction$ OR Manifestation$)))	
#5	TS=("Randomized Controlled" OR "Randomized Clinical" OR "Controlled Clinical" OR RCT OR Cohort OR "Concurrent Stud*" OR Prospect*)	
#6	#5 AND #4 AND #3 AND #2 AND #1 index=SCI-EXPANDED, SSCI	653
	Cochrane Library	
#1	(intracranial OR cerebral OR Brain OR "Basilar Artery" OR Saccular OR "Anterior Communicating Artery" OR "Posterior Communicating Artery" OR "Middle Cerebral Artery" OR "Posterior Cerebral Artery" OR "Anterior Cerebral Artery" OR "Anterior circulation" OR "Posterior circulation" OR Berry OR subarachnoid):ti,ab,kw	
#2	(aneurysm* OR hemorrhage*):ti,ab,kw	
#3	#1 AND #2	
#4	MeSH descriptor: [Intracranial Aneurysm] explode all trees	
#5	MeSH descriptor: [Subarachnoid Hemorrhage] explode all trees	

#6	#3 OR #4 OR #5	
#7	(Craniotom* OR Craniectom* OR clip* OR microsurg*):ti,ab,kw	
#8	MeSH descriptor: [Craniotomy] explode all trees	
#9	MeSH descriptor: [Microsurgery] explode all trees	
#10	#7 OR #8 OR #9	
#11	(coil* or emboli* or Embolotherap* or endovascular or Intravascular or neuroradiology or Guglielmi or Prosthes* or Implant* or Endoprosthes*):ti,ab,kw	
#12	MeSH descriptor: [Embolization, Therapeutic] explode all trees	
#13	MeSH descriptor: [Prostheses and Implants] explode all trees	
#14	#11 OR #12 OR #13	
#15	(Death OR Mortalit* OR Fatalit* OR Morbidit* OR Dependenc* OR Disabl* OR outcome OR Efficacy OR Effectiveness OR Prognos* OR "Glasgow Outcome Scale" OR gos OR "Modified Rankin Score" OR mrs OR Complication* OR Vasospasm$ OR Angiospasm$ OR "Vascular Spasm$" OR "Cerebrovascular Spasm$" OR Ischemi* OR Infarct* OR Stroke$ OR "Intracranial infection" OR Hydrocephal* OR Rebleed OR Obliteration$ OR Occlusion$ OR Recanalisation OR "Hospital stay$" OR "Length of Stay" OR "Stay Length$" OR (Neurologic* AND (deficit$ OR Symptom$ OR Sign$ OR Dysfunction$ OR Manifestation$))):ti,ab,kw	
#16	MeSH descriptor: [Death] explode all trees	
#17	MeSH descriptor: [Mortality] explode all trees	
#18	MeSH descriptor: [Morbidity] explode all trees	
#19	MeSH descriptor: [Prognosis] explode all trees	
#20	MeSH descriptor: [Glasgow Outcome Scale] explode all trees	
#21	MeSH descriptor: [Vasospasm, Intracranial] explode all trees	
#22	MeSH descriptor: [Ischemia] explode all trees	
#23	MeSH descriptor: [Neurologic Manifestations] explode all trees	
#24	MeSH descriptor: [Infarction] explode all trees	
#25	MeSH descriptor: [Stroke] explode all trees	
#26	MeSH descriptor: [Hydrocephalus] explode all trees	
#27	MeSH descriptor: [Length of Stay] explode all trees	
#28	#15 OR #16 OR #17 OR #18 OR #19 OR #20 OR #21 OR #22 OR #23 OR #24 OR #25 OR #26 OR #27	
#29	("Randomized Controlled" or "Randomized Clinical" or "Controlled Clinical" or RCT or Cohort or "Concurrent Stud*"or Prospect*):ti,ab,kw	
#30	MeSH descriptor: [Randomized Controlled Trial] explode all trees	
#31	MeSH descriptor: [Cohort Studies] explode all trees	
#32	#29 OR #30 OR #31	
#33	#6 AND #10 AND #14 AND #28 AND #32	290
	Pubmed	
#1	Search ((((((((coil*[Title/Abstract]) OR emboli*[Title/Abstract]) OR Embolotherap*[Title/Abstract]) OR endovascular[Title/Abstract]) OR Intravascular[Title/Abstract]) OR neuroradiology[Title/Abstract]) OR Guglielmi[Title/Abstract]) OR Prosthes*[Title/Abstract]) OR Implant*[Title/Abstract]) OR Endoprosthes*[Title/Abstract]	
#2	Search (((((Craniotom*[Title/Abstract]) OR Craniectom*[Title/Abstract]) OR clip*[Title/Abstract]) OR microsurg*[Title/Abstract]))	

#3	Search (((Neurologic*[Title/Abstract]) AND ((((((deficit*[Title/Abstract]) OR Symptom*[Title/Abstract]) OR Sign[Title/Abstract]) OR Signs[Title/Abstract]) OR Dysfunction*[Title/Abstract]) OR Manifestation*[Title/Abstract]))) OR (((((((((((((((((((((((((((((((((((Death*[Title/Abstract]) OR Mortalit*[Title/Abstract]) OR Fatalit*[Title/Abstract]) OR Morbidit*[Title/Abstract]) OR Dependenc*[Title/Abstract]) OR Disabl*[Title/Abstract]) OR outcome[Title/Abstract]) OR Efficacy[Title/Abstract]) OR Effectiveness[Title/Abstract]) OR Prognos*[Title/Abstract]) OR "Glasgow Outcome Scale"[Title/Abstract]) OR GOS[Title/Abstract]) OR "Modified Rankin Score"[Title/Abstract]) OR mRS[Title/Abstract]) OR Complication*[Title/Abstract]) OR Vasospasm*[Title/Abstract]) OR Angiospasm*[Title/Abstract]) OR "Vascular Spasm"[Title/Abstract]) OR "Vascular Spasms"[Title/Abstract]) OR "Cerebrovascular Spasm"[Title/Abstract]) OR "Cerebrovascular Spasms"[Title/Abstract]) OR Ischemi*[Title/Abstract]) OR Infarct*[Title/Abstract]) OR Stroke*[Title/Abstract]) OR infect*[Title/Abstract]) OR Hydrocephal*[Title/Abstract]) OR Rebleed[Title/Abstract]) OR Obliteration*[Title/Abstract]) OR Occlusion*[Title/Abstract]) OR Recanalisation*[Title/Abstract]) OR "Hospital stay"[Title/Abstract]) OR "Hospital stays"[Title/Abstract]) OR "Length of Stay"[Title/Abstract]) OR "Stay Length"[Title/Abstract]) OR "Stay Lengths"[Title/Abstract])
#4	Search (((aneurysm*[Title/Abstract]) OR hemorrhage*[Title/Abstract])) AND ((((((((((((((intracranial[Title/Abstract]) OR cerebral[Title/Abstract]) OR Brain[Title/Abstract]) OR "Basilar Artery"[Title/Abstract]) OR Saccular[Title/Abstract]) OR "Anterior Communicating Artery"[Title/Abstract]) OR "Posterior Communicating Artery"[Title/Abstract]) OR "Middle Cerebral Artery"[Title/Abstract]) OR "Posterior Cerebral Artery"[Title/Abstract]) OR "Anterior Cerebral Artery"[Title/Abstract]) OR "Anterior circulation"[Title/Abstract]) OR "Posterior circulation"[Title/Abstract]) OR Berry[Title/Abstract]) OR subarachnoid[Title/Abstract])
#5	Search ("Intracranial Aneurysm"[Mesh]) OR "Subarachnoid Hemorrhage"[Mesh]
#6	Search ((((((((((((((((("Death"[Mesh]) OR "Mortality"[Mesh]) OR "Morbidity"[Mesh]) OR "Outcome Assessment (Health Care)"[Mesh]) OR "Glasgow Outcome Scale"[Mesh]) OR "Treatment Outcome"[Mesh]) OR "Comparative Effectiveness Research"[Mesh]) OR "Prognosis"[Mesh]) OR "Intraoperative Complications"[Mesh]) OR "Postoperative Complications"[Mesh]) OR "Vasospasm, Intracranial"[Mesh]) OR "Ischemia"[Mesh]) OR "Neurologic Manifestations"[Mesh]) OR "Infarction"[Mesh]) OR "Stroke"[Mesh]) OR "Hydrocephalus"[Mesh]) OR "Therapeutic Occlusion"[Mesh]) OR "Length of Stay"[Mesh]
#7	Search (("Embolization, Therapeutic"[Mesh]) OR "Endovascular Procedures"[Mesh]) OR "Prostheses and Implants"[Mesh]
#8	Search ((("Craniotomy"[Mesh]) OR "Microsurgery"[Mesh]))
#9	Search (((((((((((("Randomized Controlled"[Title/Abstract]) OR "Randomized Clinical"[Title/Abstract]) OR "Controlled Clinical"[Title/Abstract]) OR RCT[Title/Abstract]) OR Cohort[Title/Abstract]) OR "Concurrent Study"[Title/Abstract]) OR "Concurrent Studies"[Title/Abstract]) OR Prospect*[Title/Abstract])

#10	Search (((("Randomized Controlled Trials as Topic"[Mesh]) OR "Cohort Studies"[Mesh]) OR "Prospective Studies"[Mesh])	
#11	(#1 OR #7) AND (#2 OR #8) AND (#3 OR #6) AND (#4 OR #5) AND (#9 OR #10)	1480
	the China National Knowledge Infrastructure	
#1	(SU = ('动脉瘤'+ '出血') AND SU = ('颅内' + '脑' + '基底'+ '囊状' + '交通' + '循环' +'颅底' + '蛛网膜下腔')) AND SU =('开颅'+ '夹闭' + '显微') AND SU = ('弹簧圈' + '栓塞' + '线圈' + '血管内' + '介入' + '神经放射学' + 'Guglielmi' + '假体' + '植入物') AND SU = ('死亡' + '发病' + '依赖' + '残' + '结果' + '疗效' + '效果' + '预后' + 'GOS' + 'mrs' + 'Rankin' + '并发症' + '血管痉挛' + '缺血' + '神经功能缺损' + '梗塞' + '中风' + '颅内感染' + '脑积水' + '再出血' + '消除' + '闭塞' + '再通' + '复发' + '住院时间') AND SU=('随机对照'+'队列'+'前瞻性'+'同时性研究'+)	12
	Wanfang	
#1	主题:(("动脉瘤"+"出血")*("颅内"+"脑"+"基底"+"囊状"+"交通"+"循环"+"颅底"+"蛛网膜下腔"))*("开颅"+"夹闭"+"显微")*("弹簧圈"+"栓塞"+"线圈"+"血管内"+"介入"+"神经放射学"+"Guglielmi"+"假体"+"植入物")*("死亡"+"发病"+"依赖"+"残"+"结果"+"疗效"+"效果"+"预后"+"GOS"+"mrs"+"Rankin"+"并发症"+"血管痉挛"+"缺血"+"神经功能缺损"+"梗塞"+"中风"+"颅内感染"+"脑积水"+"再出血"+"消除"+"闭塞"+"再通"+"复发"+"住院时间")*（"随机对照"+"队列"+"前瞻性"+"同时性研究")*Date:-2019<br>限制：期刊论文、学位论文、会议论文	71
	Sinomed	
#1	(("动脉瘤"[常用字段] OR "出血"[常用字段]) AND ("颅"[常用字段] OR "脑"[常用字段] OR "基底"[常用字段] OR "囊状"[常用字段] OR "交通"[常用字段] OR "循环"[常用字段] OR "蛛网膜下腔"[常用字段])) AND ("开颅"[常用字段] OR "夹闭"[常用字段] OR "显微"[常用字段]) AND ("弹簧圈"[常用字段] OR "栓塞"[常用字段] OR "线圈"[常用字段] OR "血管内"[常用字段] OR "介入"[常用字段]) AND ("随机对照"[常用字段] OR "队列"[常用字段] OR "前瞻性"[常用字段])	153
#2	("颅内动脉瘤"[不加权:扩展] OR "蛛网膜下腔出血"[不加权:扩展] OR "颅内出血"[不加权:扩展] OR "脑出血"[不加权:扩展]) AND ("显微外科手术"[不加权:扩展]) AND ("栓塞, 治疗性"[不加权:扩展] OR "假体和植入物"[不加权:扩展]) AND ("随机对照试验(主题)"[不加权:扩展] OR "队列研究"[不加权:扩展] OR "前瞻性研究"[不加权:扩展] ）	0

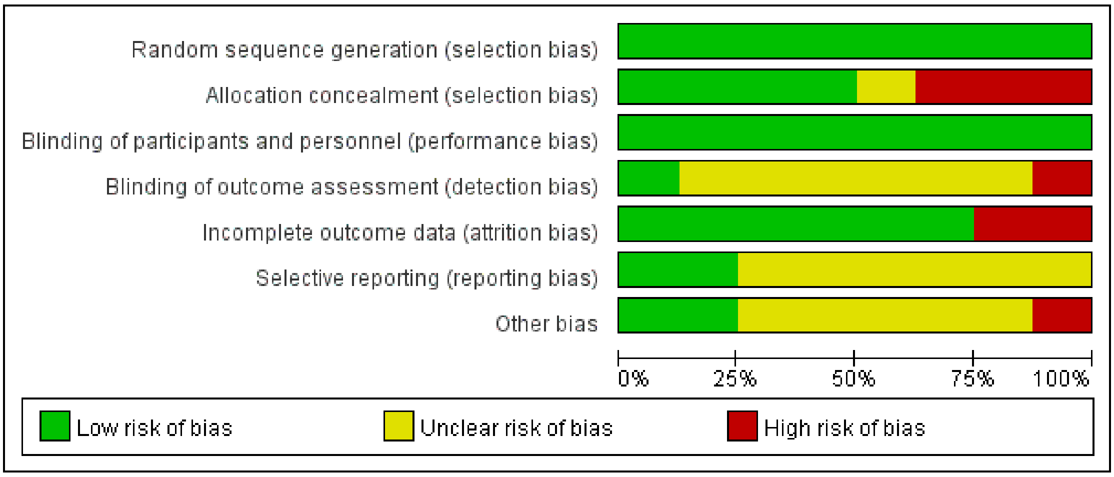

Supplemental Figure 1. The overall methodological quality of included RCTs.

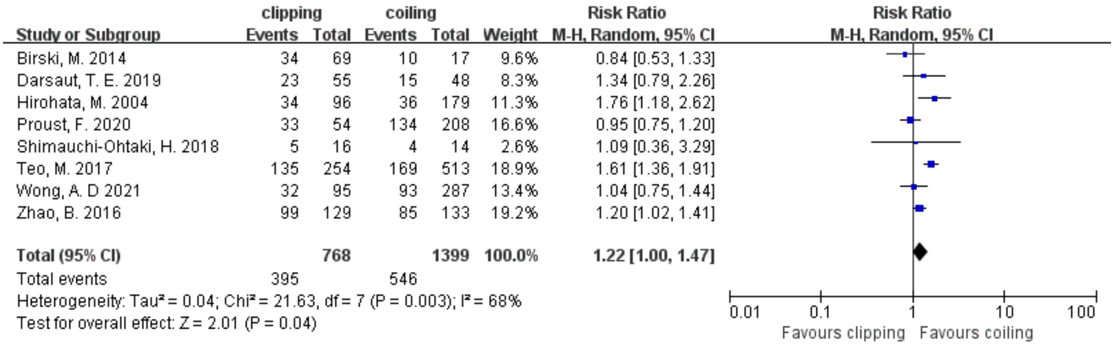

Supplemental Figure 2. Effect of clipping versus coiling on the poor outcome rate at discharge.

CI- confidence interval; M-H- Mantel– Haenszel method.

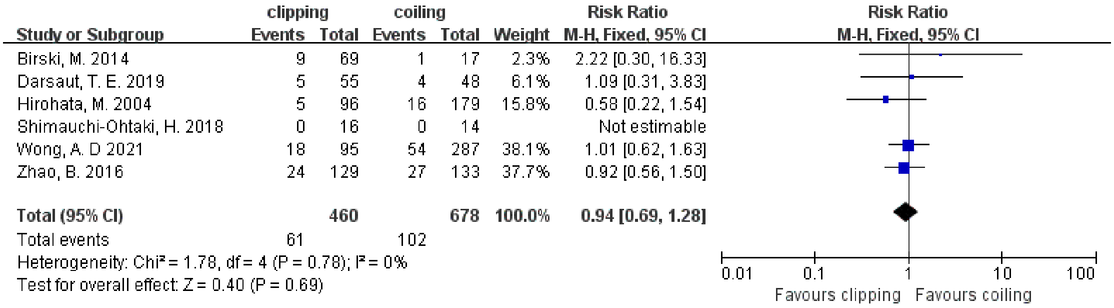

Supplemental Figure 3. Effect of clipping versus coiling on the mortality at discharge.

CI- confidence interval; M-H- Mantel– Haenszel method.

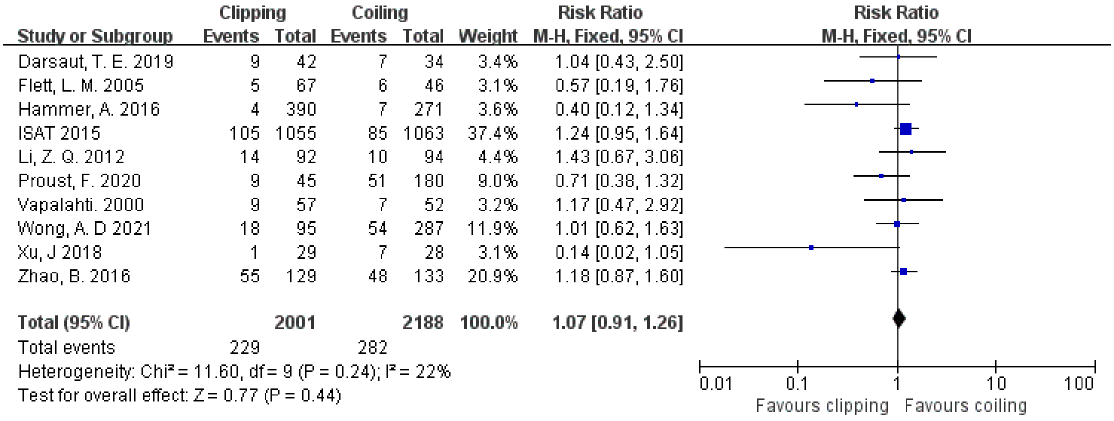

Supplemental Figure 4. Effect of clipping versus coiling on the mortality at 1-year follow-up.

ISAT- the International Subarachnoid Aneurysm Trial; CI- confidence interval; M-H- Mantel–Haenszel method.

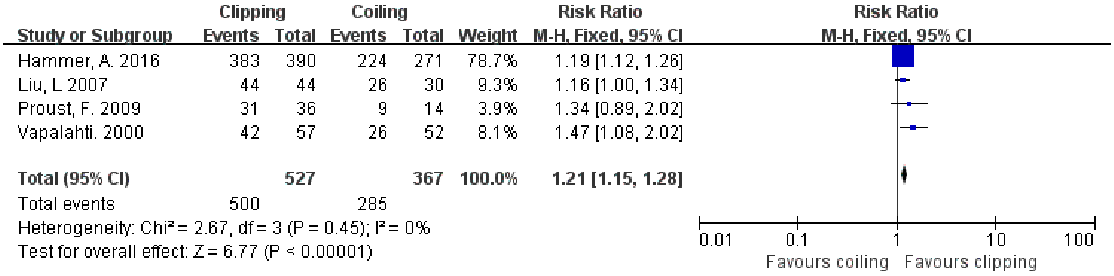

Supplemental Figure 5. Effect of clipping versus coiling on the complete aneurysm obliteration rate at discharge.

CI- confidence interval; M-H- Mantel– Haenszel method.

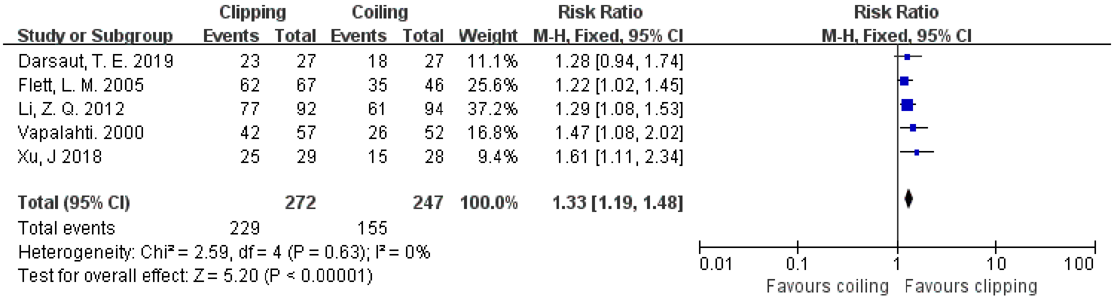

Supplemental Figure 6. Effect of clipping versus coiling on the complete aneurysm obliteration rate at 1-year follow-up.

CI- confidence interval; M-H- Mantel– Haenszel method.

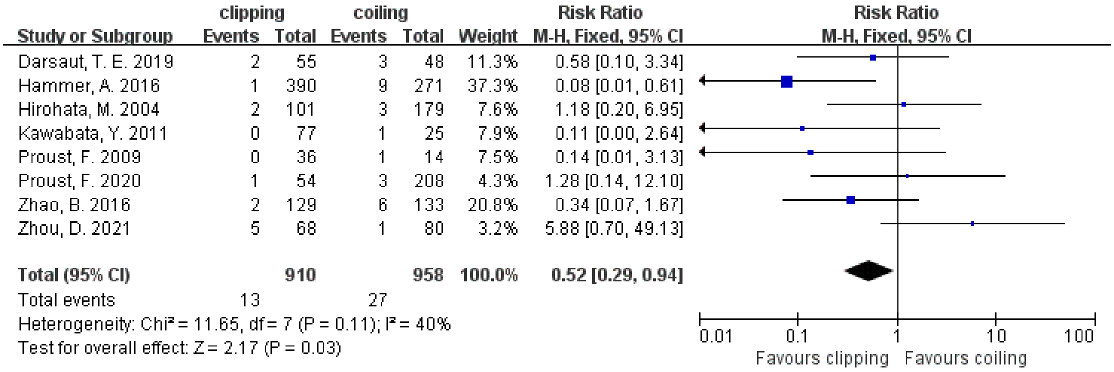

Supplemental Figure 7. Effect of clipping versus coiling on the rebleeding rate at discharge.

CI- confidence interval; M-H- Mantel– Haenszel method.

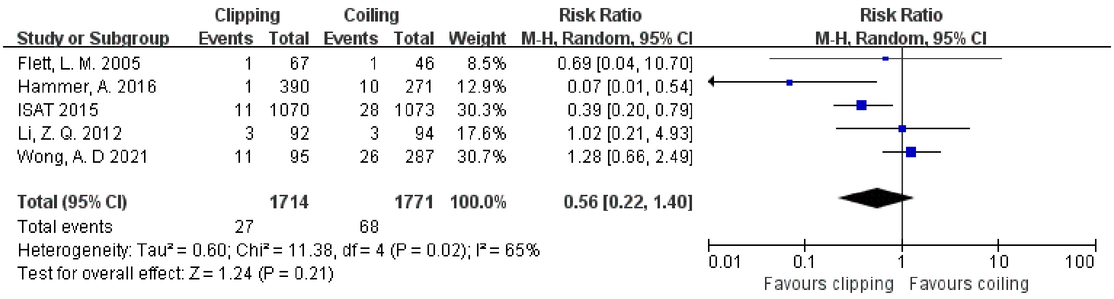

Supplemental Figure 8. Effect of clipping versus coiling on the rebleeding rate at 1-year follow-up.

ISAT- the International Subarachnoid Aneurysm Trial; CI- confidence interval; M-H- Mantel– Haenszel method.

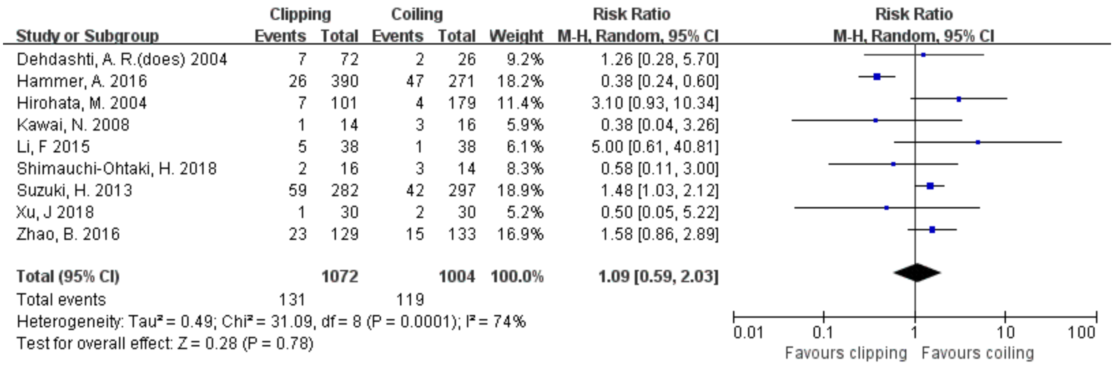

Supplemental Figure 9. Effect of clipping versus coiling on the ischemic cerebral infarction rate at discharge.

CI- confidence interval; M-H- Mantel– Haenszel method.

Study or Subgroup	Clipping Events	Clipping Total	Coiling Events	Coiling Total	Weight	Risk Ratio M-H, Random, 95% CI
Darsaut, T. E. 2019	11	27	5	27	20.9%	2.20 [0.88, 5.48]
Hammer, A. 2016	22	390	38	271	26.6%	0.40 [0.24, 0.66]
Li, Z. Q. 2012	20	92	12	94	24.6%	1.70 [0.88, 3.28]
Wong, A. D 2021	26	95	58	287	27.8%	1.35 [0.91, 2.02]
**Total (95% CI)**		**604**		**679**	**100.0%**	**1.15 [0.54, 2.44]**
Total events	79		113			

Heterogeneity: Tau² = 0.49; Chi² = 20.38, df = 3 (P = 0.0001); I² = 85%
Test for overall effect: Z = 0.36 (P = 0.72)

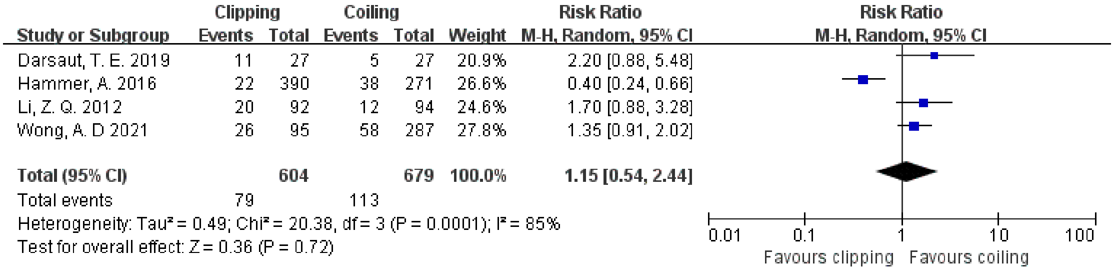

Supplemental Figure 10. Effect of clipping versus coiling on the ischemic cerebral infarction rate at 1-year follow-up.

CI- confidence interval; M-H- Mantel– Haenszel method.

Study or Subgroup	Clipping Events	Clipping Total	Coiling Events	Coiling Total	Weight	Risk Ratio M-H, Random, 95% CI
Darsaut, T. E. 2019	8	55	4	48	9.9%	1.75 [0.56, 5.44]
Dehdashti, A. R.(shunt) 2004	25	180	13	65	17.9%	0.69 [0.38, 1.27]
Koyanagi, M. 2019	41	136	22	136	20.7%	1.86 [1.18, 2.95]
Li, F 2015	6	38	2	38	6.5%	3.00 [0.65, 13.94]
Mortimer, A. M. 2016	35	66	37	69	23.4%	0.99 [0.72, 1.36]
Shimauchi-Ohtaki, H. 2018	5	16	5	14	11.4%	0.88 [0.32, 2.40]
Xu, J 2018	2	30	1	30	3.3%	2.00 [0.19, 20.90]
Zhao, B. 2016	2	129	14	133	7.0%	0.15 [0.03, 0.64]
**Total (95% CI)**		**650**		**533**	**100.0%**	**1.06 [0.68, 1.67]**
Total events	124		98			

Heterogeneity: Tau² = 0.20; Chi² = 17.68, df = 7 (P = 0.01); I² = 60%
Test for overall effect: Z = 0.26 (P = 0.79)

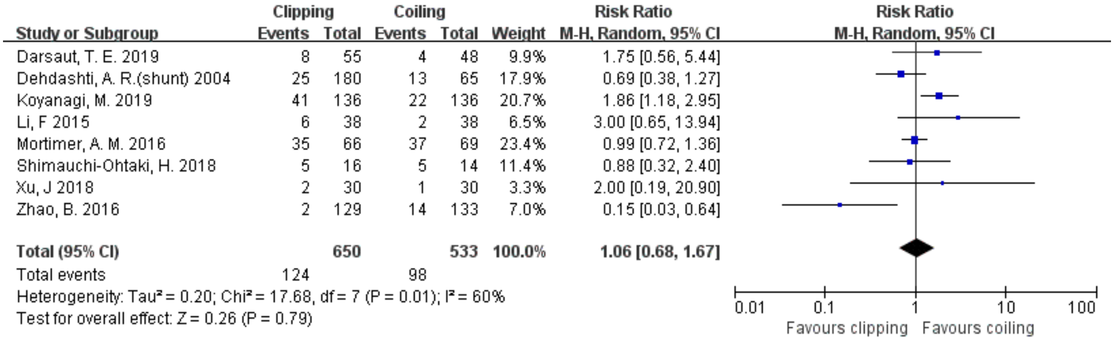

Supplemental Figure 11. Effect of clipping versus coiling on the shunt-dependent hydrocephalus rate at discharge.

CI- confidence interval; M-H- Mantel– Haenszel method.

Study or Subgroup	Clipping Events	Clipping Total	coiling Events	coiling Total	Weight	Risk Ratio M-H, Fixed, 95% CI
Bian, L. H. 2012	4	12	3	9	2.1%	1.00 [0.29, 3.39]
Darsaut, T. E. 2019	17	55	8	48	5.3%	1.85 [0.88, 3.91]
Dehdashti, A. R.(does) 2004	18	72	4	26	3.7%	1.63 [0.61, 4.36]
Groden, C. 2000	2	22	1	12	0.8%	1.09 [0.11, 10.83]
Hirohata, M. 2004	29	101	8	166	3.8%	5.96 [2.84, 12.52]
Mortimer, A. M. 2016	35	66	30	69	18.3%	1.22 [0.86, 1.73]
Shimauchi-Ohtaki, H. 2018	6	16	5	14	3.3%	1.05 [0.41, 2.70]
Suzuki, H. 2013	69	282	57	297	34.7%	1.27 [0.93, 1.74]
Wan, Z 2017	36	43	24	40	15.6%	1.40 [1.05, 1.86]
Xu, J 2018	2	30	4	30	2.5%	0.50 [0.10, 2.53]
Zhao, B. 2016	13	129	14	133	8.6%	0.96 [0.47, 1.96]
Zhou, D. 2021	3	68	2	80	1.1%	1.76 [0.30, 10.25]
**Total (95% CI)**		**896**		**924**	**100.0%**	**1.45 [1.23, 1.71]**
Total events	234		160			

Heterogeneity: Chi² = 19.89, df = 11 (P = 0.05); I² = 45%
Test for overall effect: Z = 4.38 (P < 0.0001)

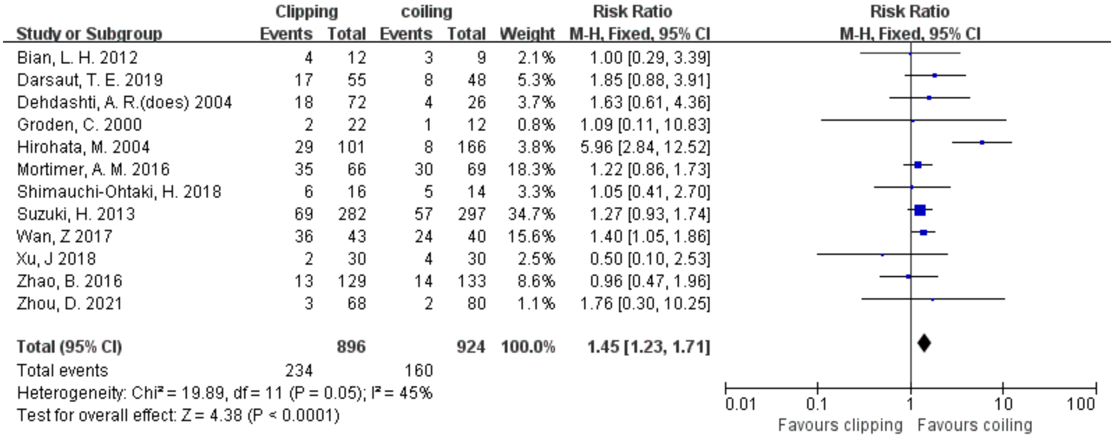

Supplemental Figure 12. Effect of clipping versus coiling on the vasospasm rate at discharge.

CI- confidence interval; M-H- Mantel– Haenszel method.

Study or Subgroup	Clipping Events	Clipping Total	Coiling Events	Coiling Total	Weight	Risk Ratio M-H, Fixed, 95% CI
**11.1.1 Fisher grade 0-2**						
ISAT 2015	44	234	28	245	3.4%	1.65 [1.06, 2.55]
Vapalahti. 2000	3	22	2	20	0.3%	1.36 [0.25, 7.34]
**Subtotal (95% CI)**		**256**		**265**	**3.6%**	**1.63 [1.06, 2.48]**
Total events	47		30			
Heterogeneity: Chi² = 0.04, df = 1 (P = 0.83); I² = 0%						
Test for overall effect: Z = 2.24 (P = 0.02)						
**11.1.2 Fisher grade 3-4**						
ISAT 2015	282	821	222	818	27.3%	1.27 [1.09, 1.47]
Vapalahti. 2000	11	35	9	32	1.2%	1.12 [0.53, 2.34]
**Subtotal (95% CI)**		**856**		**850**	**28.5%**	**1.26 [1.09, 1.45]**
Total events	293		231			
Heterogeneity: Chi² = 0.10, df = 1 (P = 0.75); I² = 0%						
Test for overall effect: Z = 3.14 (P = 0.002)						
**11.1.3 WFNS 1-3**						
Flett, L. M. 2005	23	56	9	38	1.3%	1.73 [0.90, 3.33]
ISAT 2015	291	996	208	999	25.5%	1.40 [1.20, 1.64]
Proust, F. 2020	15	38	50	152	2.5%	1.20 [0.76, 1.89]
**Subtotal (95% CI)**		**1090**		**1189**	**29.3%**	**1.40 [1.21, 1.62]**
Total events	329		267			
Heterogeneity: Chi² = 0.86, df = 2 (P = 0.65); I² = 0%						
Test for overall effect: Z = 4.61 (P < 0.00001)						
**11.1.4 WFNS 4-6**						
Flett, L. M. 2005	11	11	4	8	0.6%	1.92 [0.99, 3.72]
ISAT 2015	35	59	42	64	5.0%	0.90 [0.69, 1.19]
Proust, F. 2020	6	7	23	28	1.1%	1.04 [0.74, 1.48]
**Subtotal (95% CI)**		**77**		**100**	**6.7%**	**1.02 [0.82, 1.27]**
Total events	52		69			
Heterogeneity: Chi² = 4.22, df = 2 (P = 0.12); I² = 53%						
Test for overall effect: Z = 0.20 (P = 0.84)						
**11.1.5 Anterior cerebral and anterior communicating**						
ISAT 2015	147	534	131	533	16.1%	1.12 [0.91, 1.37]
Vapalahti. 2000	5	28	4	27	0.5%	1.21 [0.36, 4.02]
**Subtotal (95% CI)**		**562**		**560**	**16.6%**	**1.12 [0.92, 1.37]**
Total events	152		135			
Heterogeneity: Chi² = 0.01, df = 1 (P = 0.91); I² = 0%						
Test for overall effect: Z = 1.13 (P = 0.26)						
**11.1.6 Middle cerebral**						
ISAT 2015	39	139	46	162	5.2%	0.99 [0.69, 1.42]
Vapalahti. 2000	3	12	2	7	0.3%	0.88 [0.19, 4.03]
**Subtotal (95% CI)**		**151**		**169**	**5.5%**	**0.98 [0.69, 1.40]**
Total events	42		48			
Heterogeneity: Chi² = 0.02, df = 1 (P = 0.88); I² = 0%						
Test for overall effect: Z = 0.10 (P = 0.92)						
**11.1.7 Internal carotid**						
ISAT 2015	125	348	69	344	8.5%	1.79 [1.39, 2.31]
Vapalahti. 2000	3	12	3	12	0.4%	1.00 [0.25, 4.00]
**Subtotal (95% CI)**		**360**		**356**	**8.9%**	**1.76 [1.37, 2.25]**
Total events	128		72			
Heterogeneity: Chi² = 0.66, df = 1 (P = 0.42); I² = 0%						
Test for overall effect: Z = 4.44 (P < 0.00001)						
**11.1.8 Posterior circulation**						
ISAT 2015	15	34	4	24	0.6%	2.65 [1.00, 6.99]
Vapalahti. 2000	3	5	2	6	0.2%	1.80 [0.47, 6.87]
**Subtotal (95% CI)**		**39**		**30**	**0.8%**	**2.41 [1.08, 5.37]**
Total events	18		6			
Heterogeneity: Chi² = 0.22, df = 1 (P = 0.64); I² = 0%						
Test for overall effect: Z = 2.15 (P = 0.03)						
**Total (95% CI)**		**3391**		**3519**	**100.0%**	**1.31 [1.22, 1.42]**
Total events	1061		858			
Heterogeneity: Chi² = 26.13, df = 17 (P = 0.07); I² = 35%						
Test for overall effect: Z = 6.96 (P < 0.00001)						
Test for subgroup differences: Chi² = 19.33, df = 7 (P = 0.007), I² = 63.8%						

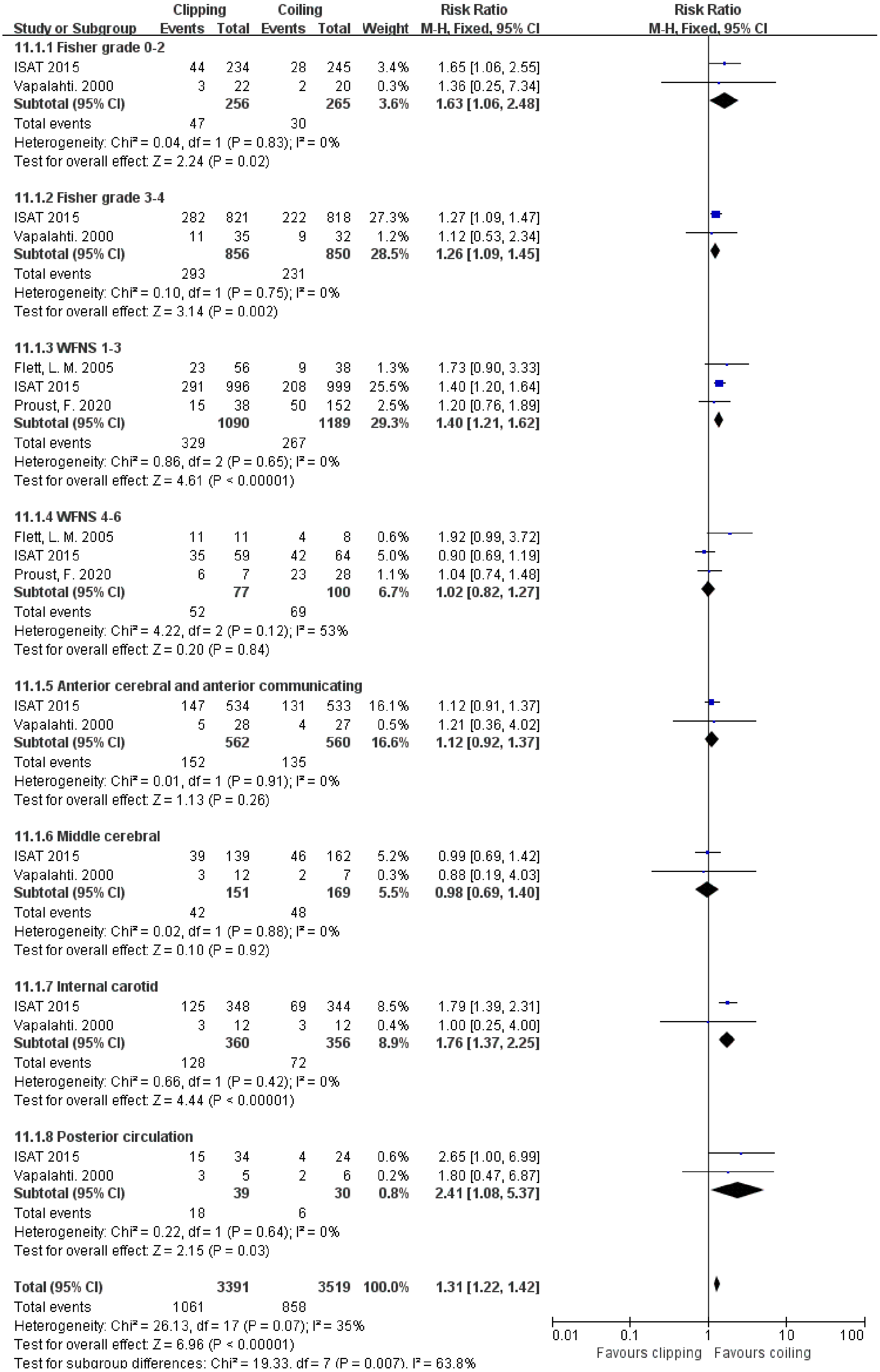

Supplemental Figure 13: The subgroup analyses on the 1-year poor outcome rate.

ISAT- the International Subarachnoid Aneurysm Trial; CI- confidence interval; M-H- Mantel–Haenszel method.
